# The Design and Optimization of a Compressive-Type Vector Sensor Utilizing a PMN-28PT Piezoelectric Single-Crystal

**DOI:** 10.3390/s19235155

**Published:** 2019-11-25

**Authors:** Hong Goo Yeo, Junhee Choi, Changzhu Jin, Seonghun Pyo, Yongrae Roh, Hongsoo Choi

**Affiliations:** 1 Department of Robotics Engineering, DGIST, Daegu 42988, Korea; hgyeo@dgist.ac.kr (H.G.Y.); jchoi46@dgist.ac.kr (J.C.); yustchang@dgist.ac.kr (C.J.); 2 DGIST-ETH Microrobot Research Center, DGIST, Daegu 42988, Korea; 3 School of Mechanical Engineering, Kyungpook National University, Daegu 41566, Korea; nasgool@naver.com (S.P.); yryong@knu.ac.kr (Y.R.)

**Keywords:** single-crystal accelerometer, compressive-type vector sensor, receiving voltage sensitivity, dipole beam pattern, cardioid beam pattern

## Abstract

Underwater sensors that detect the distance and direction of acoustic sources are critical for surveillance monitoring and target detection in the water. Here, we propose an axial vector sensor that utilizes a small (~1 cm^3^) compressive-type piezoelectric accelerometer using piezoelectric single crystals. Initially, finite element analysis (FEA) was used to optimize the structure that comprised piezoelectric Pb(Mb_1/3_Nb_2/3_)O_3_-28%PbTiO_3_ single crystals on a tungsten seismic mass. The receiving voltage sensitivity (RVS) was enhanced through geometric optimization of the thickness and sensing area of the piezoelectric material and the seismic mass. The estimated maximum RVS of the optimized vector sensor was −212 dB. FEA simulations and practical measurements were used to verify the directivity of the vector sensor design, which exhibited a dipole pattern. The dipole beam pattern was used to obtain cardioid patterns using the simulated and measured results for comparison. The results clearly showed the feasibility of using the proposed piezoelectric single-crystal accelerometer for a compressive-type vector sensor.

## 1. Introduction

The detection of underwater targets, such as submarines and torpedoes, requires measurement of the pressure and vector components of an acoustic source. Underwater acoustic detection techniques are typically classified as active or passive detections. In the case of active systems, it contains not only a receiver for perceiving the reflection echoes from objects, but also a transmitter for emitting coded acoustic waves with multiple frequencies and various intervals. Passive acoustic detection systems only including receivers such as hull-mounted sonar (HMS) and towed array sonar system (TASS) identify sounds generated by submarines or torpedoes. Most TASS systems used for underwater target detection comprise a line array of omnidirectional cylindrical hydrophones. However, the hydrophone line array may be several kilometers long, which results in a degree of sensor-positioning uncertainty [1].

The limitations of TASS, such as poor vessel maneuverability, can be overcome using an underwater sensor network system, which can easily extend the detection area using multiple sensor nodes that reduce detection errors [2]. The number of sensors required by wireless underwater sensor networks to detect or monitor targets depends on the area to be tracked. An acoustic vector sensor is a versatile device that measures the pressure and vector component of acoustic wave simultaneously, without any need for the complex signal processing required when using multimode vector hydrophones [3,4].

Acoustic vector sensors typically operate on the principle of pressure–pressure or pressure–velocity measurements [5]. Pressure–pressure sensors can be used to estimate particle velocity by measuring the pressure gradient between two closely spaced sensors [6,7,8]. However, the performance of gradient sensors is negatively affected by problems such as finite difference approximation, scattering, diffraction, and instrument phase mismatch [9]. In contrast, inertial pressure–velocity-based sensors utilize an accelerometer to sense the particle velocity directly [10,11,12]. An axial accelerometer is most sensitive along a specific direction in a three-dimensional (3-D) axis and is relatively insensitive to forces along the other two axes [5,9]. Therefore, a dipole beam pattern can be produced along the dominant accelerometer axis [13,14] by using an axial accelerometer. Combining the dipole beam pattern with that of an omnidirectional sensor having the same sensitivity in all the directions yields a cardioid beam pattern to provide directional information of an acoustic source [15,16,17].

Since the 2000s, researchers have been studying inertia-type vectors by combining an omnidirectional hydrophone with accelerometers to produce dipole and cardioid beam patterns [7,18,19]. Shipps et al. studied tri-vector hydrophones that were mounted using Pb(Mb_1/3_Nb_2/3_)O_3_-PbTiO_3_ (PMN-PT) crystals with no seismic mass inside a PZT ring omnidirectional hydrophone used for the towed-array application. Although the structure of vector sensor was not clearly described, the device met the Navy’s requirement (i.e., dipole directivity patterns, frequency range of 3 Hz to 7 kHz, hydrophone output sensitivity of −174 dB re 1 V/μPa) with a four-channel amplifier mounted inside the vector housing [13]. Pyo et al. developed a shear-thickness type vector hydrophone using Pb(In_1/2_Nb_1/2_)O_3_-Pb(Mg_1/3_Nb_2/3_)O_3_-PbTiO_3_ (PIN-PMN-PT) crystals poled along [011]_c_ [18]. For the vector hydrophone with thickness shear mode (or *d*_15_ mode), dual piezoelectric single crystals were mounted on a vertical metal base with dual seismic mass to improve the sensitivity (i.e., the minimum RVS of −201.4 dB). However, the structural features limit the weight of the masses and piezoelectric elements due to the lack of stability to support themselves with a vertical base [18]. On the other hand, there has been limited investigation of vector sensors, especially utilizing the compressive-type accelerometer. Compressive-mode vector sensors are simple in design, and can be easily used in various applications for mechanical and structural monitoring [20,21]. If the vector sensors developed here are aligned with the X, Y, and Z axes inside an omnidirectional hydrophone, they could be used to measure the directivity of an acoustic source for underwater sensor nodes.

Here, a compressive mode accelerometer is used to obtain excellent reception sensitivity and a dipole beam pattern based on analysis of the vector sensor structure, i.e., the geometry of the piezoelectric material and seismic mass. Typically, a vector sensor constructed using conventional PZT ceramics would be large, and the sensitivity cannot be better than a sensor made by a piezoelectric single crystal. The use of Pb(Mb_1/3_Nb_2/3_)O_3_-28%PbTiO_3_ (PMN-28PT) crystals allows a vector sensor size to be greatly reduced, with high sensitivity and low noise [18]. First, finite element analysis (FEA) is used to optimize the design of the PMN-28PT piezoelectric single crystal and tungsten seismic mass of the vector sensor. Then, it is shown that modifying the single vector sensor geometry allows for the estimation of the sensitivity and dipole mode beam pattern. A three-dimensional (3-D) cardioid was constructed to show the potential to find a directivity pattern in 3-D using the FEA simulations of the vector sensors in X, Y, and Z directions. Finally, the performance of the fabricated vector sensor prototype, and the simulation results are compared using the cardioid beam patterns to ensure the validity of the vector sensor design.

## 2. FEA Model of the Vector Sensor

### 2.1. Accelerometer Design

**Figure 1:**
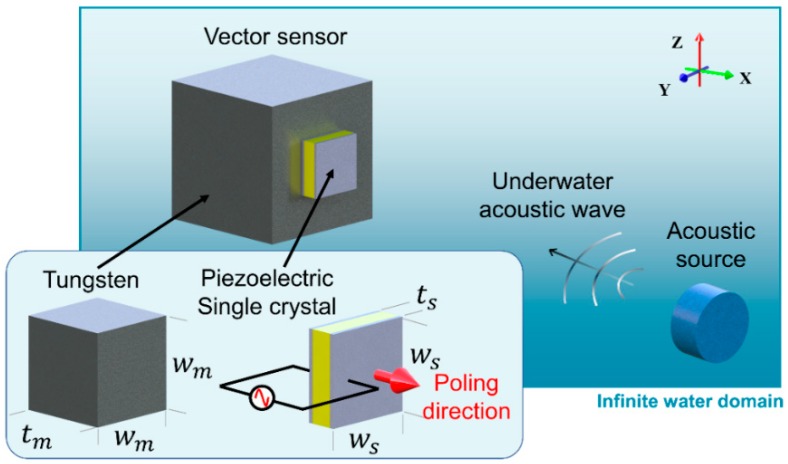
An underwater vector sensor and its design parameters.

The geometric design of a compressive accelerometer has a significant impact on the sensitivity of the resulting vector sensor. An FEA model was developed in COMSOL Multiphysics 5.4 (COMSOL Inc., Burlington, MA, USA) and used to investigate the effect of each design parameter. As shown in Figure 1, the FEA model comprised an underwater acoustic source and a compressive accelerometer. The basic structural components of the accelerometer are a piezoelectric material and a seismic mass that are both constructed from a square plane (*w* × *w*) with a thickness (*t*). The components act as a single body, and no acoustic wave is applied to the contact surface between the seismic mass and the piezoelectric material. The square frontal plane of the piezoelectric material is physically fixed, but the acoustic wave is still applied to the surface. In Figure 1, the poling and voltage output of the piezoelectric single crystal are aligned with the X-axis, which is the thickness direction of the vector sensor. The strain–charge constitutive relation was applied to the piezoelectric material to obtain the voltage output. The material properties of the PMN-28PT single crystal and tungsten presented in Table 1 and Table 2 were applied to the piezoelectric material and seismic mass, respectively [22]. The superscripts *E* and *T* represent a constant electric field and stress, respectively. *ε*_0_ is the permittivity of free space. The accelerometer design parameters are given in Table 3. For ease of fabrication, the minimum piezoelectric single crystal width (*w*_s_) was set to 2.5 mm, and the maximum *w*_s_ was set to 5 mm. The thickness range of the piezoelectric single crystal (*t_s_*) was varied from 0.1 mm to 1 mm. The tungsten mass should be larger than the piezoelectric material, so the width (*w_m_*) and thickness (*t_m_*) were varied from 5 to 10 mm and 1 to 10 mm, respectively. These size constraints were selected as the X-, Y-, and Z-axis vector sensors should be installed within an omnidirectional hydrophone with 60 mm in diameter. Therefore, the maximum dimension of a single accelerometer must be less than 10 mm so that three axial accelerometers can be installed within the omnidirectional hydrophone.

As shown in Figure 1, an acoustic source with a diameter 20 mm was located in the far-field region such that *L* > *D*^2^/4*λ*, where *L* is the distance between the source and the accelerometer, *D* is the diameter of the acoustic source, and *λ* is the wavelength [23]. An acoustic wave with a pressure of 1.5 MPa and a frequency of interest was emitted in the vertical direction, normal to the accelerometer plane. Infinite boundary conditions were applied to all the water surfaces to prevent acoustic reflection at the physical domain boundaries. The dimensions of the hexagonal mesh elements in Figure 1 were less than 0.1% of the wavelength; such a fine mesh results in a sufficient accuracy for analysis [15].

The acoustic pressure exerted on the piezoelectric material induces a mechanical vibration and generates a piezoelectric voltage output, which is derived from the strain of the piezoelectric element. The output voltage depends on the structural parameters; therefore, the sensitivity was analyzed for each structural parameter. There are several methods to evaluate the sensitivity of a vector sensor. First, the sensitivity can be given by the voltage output relative to the acoustic wave acceleration [16]. The sensitivity of the acoustic wave acceleration (*M*_a_) is expressed as *M*_a_ = *V*_out_/*a*, where *a* is the acceleration of the acoustic wave. Since the acceleration *a* equals *ωP*_i_/*ρc*, where *ω* is the angular frequency of the acoustic wave, *ρ* is the density of the medium, and *c* is the sound velocity in medium, *M*_a_ can be rewritten as the pressure sensitivity such that *M_a_* = (*ρc*/*ω*)*M*_p_, where *M*_p_ = *V*_out_/*P*_i_. It is noteworthy that since our sensor responds only to the displacement in the thickness direction, the pressure sensitivity (*M*_p_) can also be given as *M*_p_ = *g*_33_*t*, where *g*_33_ is the longitudinal piezoelectric voltage coefficient (~ *d*_33_/$\varepsilon_{33}^{T}$) and *t* is the element thickness. It is customary to express the acoustic performance of a vector sensor as receiving voltage sensitivity (RVS) in decibels as dB: 1 V/μPa [16]. Since *M*_p_ < 1 for all dimensions of thickness, the RVS is a scalar negative value.

(1) $${\mathrm{RVS}\;=\;20\log(M}_{p}\;\times \;10^{-6})\;(\mathrm{dB}\;@\;1\;V/\mu \mathrm{Pa})$$

**Figure 2:**
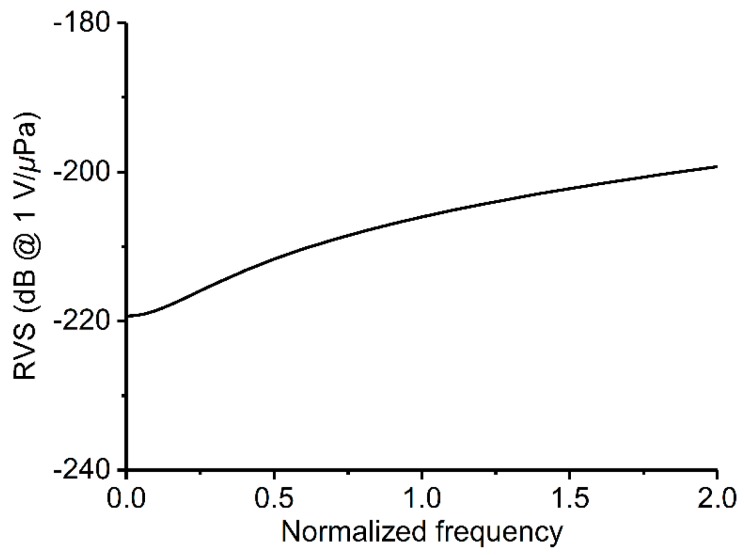
The simulated receiving voltage sensitivity (RVS) spectrum of an accelerometer comprising a piezoelectric material and seismic mass of 5 × 5 × 1 mm^3^ and 10 × 10 × 10 mm^3^, respectively.

A typical accelerometer RVS spectrum calculated using the maximum-size FEA model is shown in Figure 2. There is no singular resonant point within the defined frequency range (0.001*f*_0_–2*f*_0_). The RVS increases with frequency, and the minimum RVS occurs at the lowest frequency.

**Figure 3:**
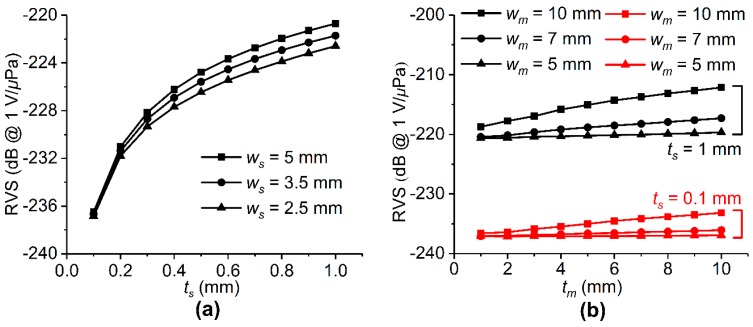
Simulation of the RVS for various **(a)** piezoelectric single crystal and **(b)** seismic mass widths (*w_s_* and *w_m_*, respectively).

The effect of piezoelectric single crystal geometry was investigated by calculating the accelerometer RVS without a seismic mass. As shown in Figure 3a, *t_s_* has a significant effect on the RVS value, as a logarithmic increase in RVS is observed when *t_s_* is increased. For *w_s_* = 5 mm, the RVS was approximately −236 and −221 dB when *t_s_* was 0.1 and 1 mm, respectively. Therefore, RVS increased by approximately 15 dB with a 0.9 mm difference in thickness. However, when *t_s_* = 1 mm, an increase of only 1.5 dB RVS was observed when *w_s_* was increased from 2.5 to 5 mm. This suggests that *t_s_* is a more crucial piezoelectric element design parameter compared to *w_s_*. The significance of the piezoelectric material thickness was further verified by adding a seismic mass. Figure 3b shows the RVS spectrum of the accelerometer when *w_s_* = 2.5 mm. For the *t_s_* = 0.1 mm (red lines) and 1 mm (black lines), the RVS increased as the seismic mass volume was increased, i.e., as a greater force was transferred to the piezoelectric element. The RVS also increased with the piezoelectric single crystal thickness. When *w_m_* and *t_m_* = 10 mm, the RVS was only −232.5 dB when *t_s_* = 0.1 mm, but it increased to −212.5 dB when *t_s_* was increased to 1 mm. Hence, increasing the piezoelectric single crystal thickness is necessary to produce an accelerometer with higher RVS.

**Figure 4:**
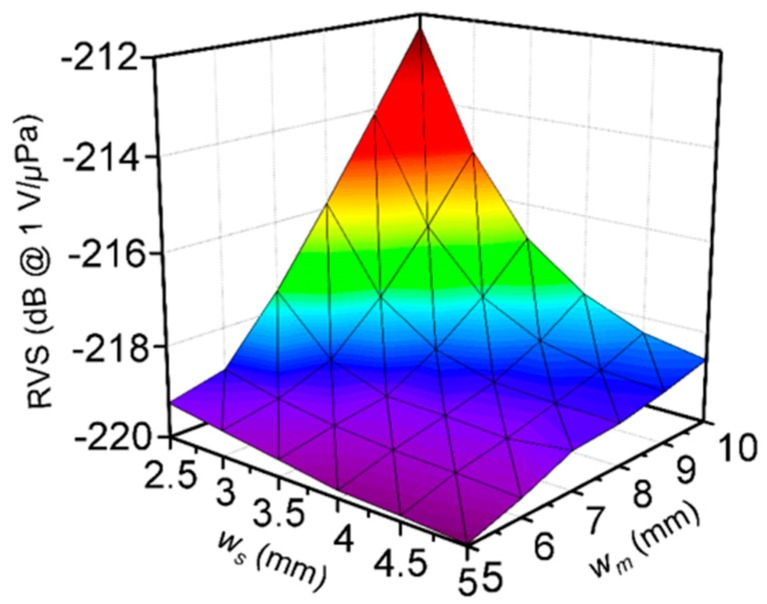
Simulation of the RVS for various piezoelectric single crystal and seismic mass widths (*w_s_* and *w_m_*, respectively).

As shown in Figure 3a, it should be noted that the area of the piezoelectric element (*w_s_* × *w_s_*) has little effect on the piezoelectric single accelerometer design, as the effect on the RVS is insignificant. However, the area is of great importance when a seismic mass is added. Therefore, the effect of *w_s_* variation was analyzed. Figure 4 presents the RVS spectrum of the accelerometer when *t_s_* and *t_m_* were maximized (1 and 10 mm, respectively). When *w_s_* and *w_m_* = 5 mm, the RVS was −220 dB, and only increased by 1.5 dB to −218.5 dB when *w_m_* was increased to 10 mm. In addition, RVS increased by only 0.5 dB, to −219.5 dB, when *w_s_* was decreased to 2.5 mm and *w_m_* was fixed at 5 mm. These minor changes in RVS may be of little importance to the accelerometer design. However, RVS increased greatly when both *w_s_* and *w_m_* were altered simultaneously. When *w_m_* = 10 mm and *w_s_* = 2.5 mm, the RVS increased by approximately 8 dB to −212 dB. It is believed that the stress exerted on the piezoelectric single crystal increases as the mass size is increased or the piezoelectric single crystal size is decreased, thereby generating a greater voltage output. Therefore, the width of the piezoelectric single crystal and the mass are important factors in the accelerometer design.

The accelerometer RVS has been analyzed by varying its dimensions as abovementioned parameters. The largest RVS was identified when the piezoelectric single crystal and seismic mass dimensions were 2.5 × 2.5 × 1 mm^3^ and 10 × 10 × 10 mm^3^, respectively, in consideration of the omnidirectional hydrophone. The receiving beam patterns were analyzed to verify their suitability for use as a vector hydrophone. Beam pattern analysis was conducted by exciting the accelerometer with an acoustic wave that was emitted from a circular acoustic source with a pressure of 1.5 MPa at a frequency of interest. The acoustic source was positioned in the far-field region, and rotated around the accelerometer from 0° to 360° at 10° intervals. The accelerometer receiving beam pattern was calculated by normalizing the voltage output.

(2) $$\mathrm{Beam}\;\mathrm{pattern}\;=\;20\log\frac{V_{out}}{V_{max}}\;\left(\mathrm{dB}\right)$$

**Figure 5:**
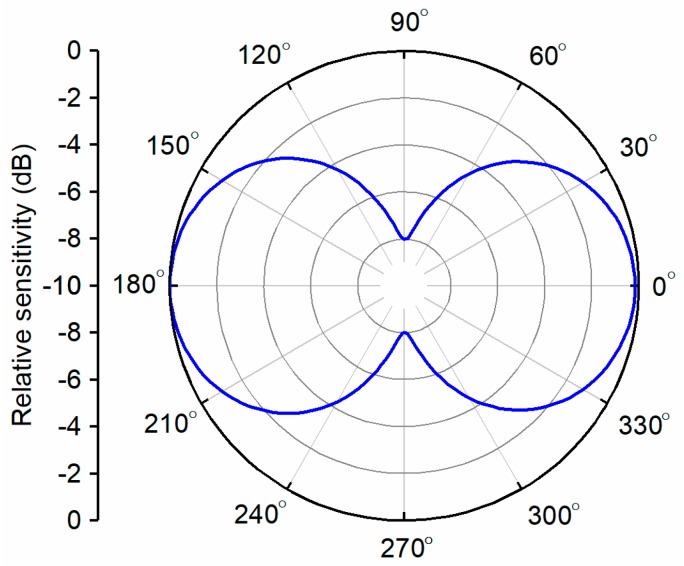
The simulated dipole beam patterns when the seismic mass size is 10 × 10 × 10 mm^3^.

Figure 5 shows the optimized accelerometer beam pattern for the piezoelectric element and seismic mass dimensions of 2.5 × 2.5 × 1 mm^3^ and 10 × 10 × 10 mm^3^, respectively. An apparent beam pattern with a sensitivity range of 8 dB was calculated and can be used to develop an accelerometer design with a clear dipole beam pattern and high RVS.

### 2.2. Vector Hydrophone

Typically, a dipole beam pattern from an accelerometer should be combined with an omnidirectional beam pattern to identify the direction of incoming acoustic waves. A vector hydrophone constructed can be realized by combining an accelerometer and omnidirectional hydrophone. This structural integration can produce a cardioid beam pattern that can be used to measure acoustic sources along a specified vector.

A cardioid beam pattern can be achieved by selectively adding and subtracting normalized dipole and omnidirectional beam patterns [8]. Here, an omnidirectional hydrophone was simulated using COMSOL Multiphysics 5.4 to obtain an omnidirectional beam pattern. A hollow spherical piezoelectric hydrophone was designed so that the accelerometers could be assembled within the hydrophone. A 1.5 MPa acoustic wave was generated at a frequency of interest and transmitted toward the geometrical centroid of hydrophone in an infinite water domain. The acoustic source was located in the far-field region and rotated around the hydrophone from 0° to 360° at 10° intervals. The diameter of the modeled hydrophones is selected as 60 mm, and the thickness was set to 2 mm. A PZT-4D was selected as the material for hydrophone, and its material properties are listed in Table 4 [24]. The poling direction was parallel to the voltage output.

**Figure 6:**
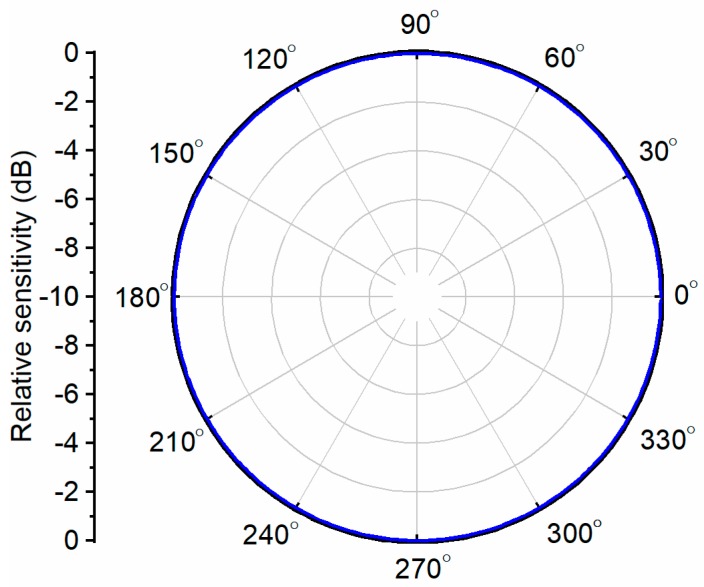
The simulated omnidirectional beam pattern of a spherical hydrophone.

The spherical hydrophone beam pattern was obtained using Equation (2), as shown in Figure 6. The omnidirectional hydrophone produces the same piezoelectric voltage output for acoustic waves incident from any direction. Therefore, a circular omnidirectional beam pattern was constructed so that the hydrophone could detect the acoustic pressure magnitude.

**Figure 7:**
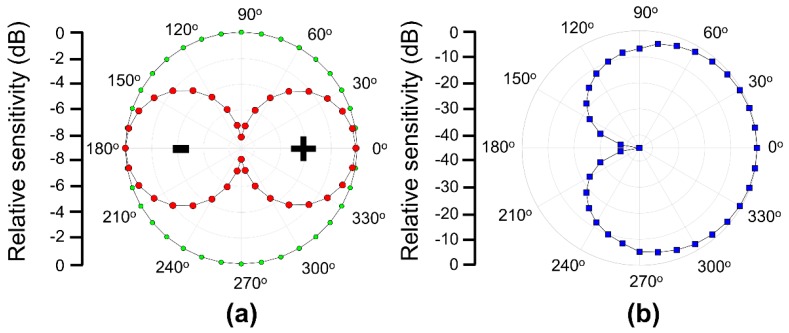
(**a**) The simulated omnidirectional hydrophone (green dots) and dipole graph (red dots) sensitivity generated by a PZT-4D and PMN-28PT. (**b**) The two-dimensional (2-D) cardiac pattern generated by combining the omnidirectional and dipole patterns shown in (**a**).

The normalized directivity of the omnidirectional hydrophone is equal in all directions, whereas the dipole has varying directivity in a different direction, as shown in Figure 7a. As the positive half of the dipole has been defined as the same sign to the corresponding omnidirectional beam, they are summed to double the amplitude. Alternatively, the polarity of the negative half of the dipole cancels the corresponding omnidirectional beam, which as a result produces a cardioid pattern. The reconstructed cardioid beam pattern is shown in Figure 7b. The difference between the frontal (0°) and minimum (180°) sensitivity is approximately 45 dB, resulting in a clear directivity along the front face of the vector hydrophone.

**Figure 8:**
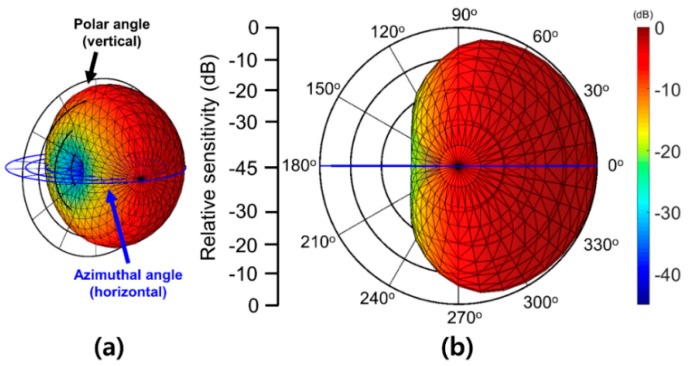
The synthesized three-dimensional (3-D) cardioid directivity pattern calculated using finite element analysis (FEA) simulation based on optimized vector sensor parameters. (**a**) The oblique view of the 3-D cardioid directivity pattern and (**b**) the normal angle view (orthogonal to polar angle plane) of one side of the reconstructed 3-D cardioid directivity (blue line: azimuthal angle plane).

The 3-D cardioid pattern of the vector sensors can be used to more accurately evaluate the 3-D directivity of an acoustic source. A 3-D dipole pattern was constructed by adding an orthogonal rotation, such as a polar angle, to the FEA model. Then, the 3-D cardioid pattern was reconstructed using the method illustrated in Figure 7 in two other directions. The resulting 3-D cardioid pattern produced using the FEA simulation is shown in Figure 8. This semi-directional pattern has the highest sensitivity on its dominant axis, and the sensitivity difference for φ = 0° and φ = 180° are 45 dB, which is in agreement with the 2-D cardioid simulation.

## 3. Sensor Fabrication and Experiment Results

### 3.1. Fabrication and Characterization of Vector Sensor

Single PMN-28PT crystals with a high piezoelectric coefficient (*d*_33_ = 1000−1200 pC/N) and high-density tungsten (17.8 g/cm^3^) were used for fabrication. PMN-28PT piezoelectric single crystals were grown to have a tetragonal symmetry of 4 mm with [001] orientation along thickness, by following the Bridgman method, were acquired from iBULe Photonics Co., Ltd. (Incheon, Korea) [22]. Following the design parameter optimization process discussed previously, a single vector sensor was fabricated using a 1-mm-thick square-shaped PMN-28PT single crystal with an active area of 6.25 mm^2^, and a cubic tungsten mass with an area of 100 mm^2^. However, practically, the small area of PMN-28PT single crystal was not strongly affixed to the relatively large volume (1000 mm^3^) and heavy (17.8 g) seismic mass (i.e., the area ratio between the piezoelectric single crystal and the mass was 1:16). The typical compression acceleration sensor manufacturing method, in which a tie bolt is used to bond the mass and piezoelectric elements together through a center hole, is not suitable for use with small piezoelectric component [20,21]. This problem was overcome by using an elastic body support layer with a very low elastic modulus around the piezoelectric component.

**Figure 9:**
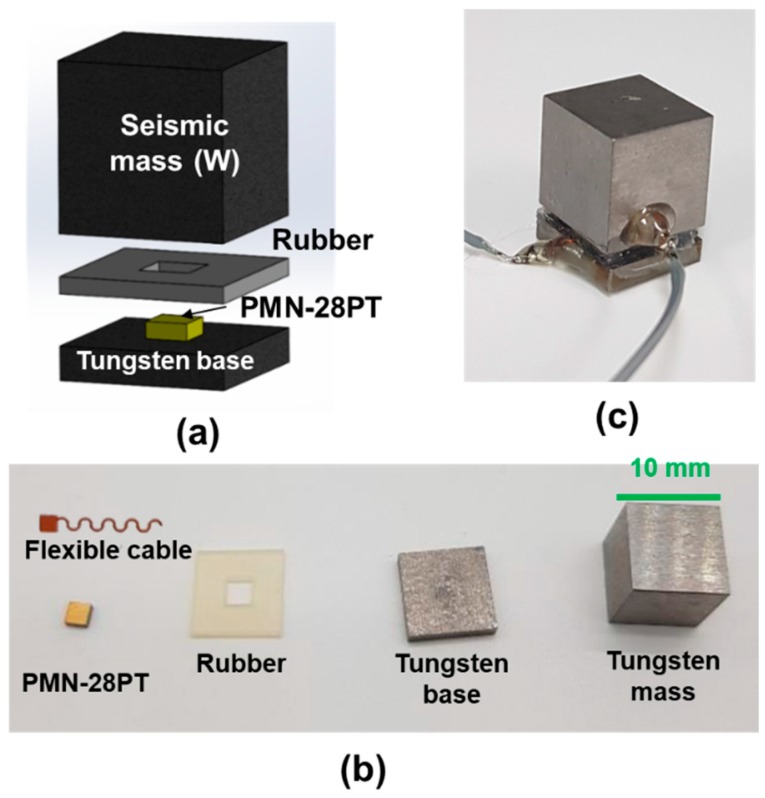
(**a**) Schematic illustration of the vector sensor. (**b**) A photograph of the sensor components. (**c**) A photograph of the fabricated single vector sensor after cabling.

Figure 9 shows the prototype vector sensor components, including PMN-28PT, a tungsten base, a seismic mass, a flexible Cu cable, and a flexible rubber layer (VisiJet CF-BK; tensile modulus: 2.2 MPa; 3D Systems, Rock Hill, SC, USA) in the form of a square ring manufactured using a 3-D printer (ProJet MJP 5500X; 3D Systems). Figure 9a presents a schematic illustration of the prototype vector sensor. Top and bottom electrodes (Au/Cr, 200 nm/25 nm) were patterned on both sides of the PMN-28PT. Au/Cr-coated PMN-28PT single-crystals were polarized along the [001] axis in a 3-kV/cm electrical field at room temperature for 30 min. Cu layer (20 μm) was spin-coated with a 5-μm-thick layer of polyimide, and cut using a laser engraving machine (VLS4.60; Universal Laser System, Scottsdale, AZ, USA) to produce a flexible cable, as shown in Figure 9b. A pair of flexible cables was used to form electrical connections, and were attached to the top and bottom electrodes using silver paste (ELCOAT A-201, CANS, Tokyo, Japan). The PMN-28PT and flexible cable were bonded to the tungsten base (10 × 10 × 2 mm^3^) using an ethyl cyanoacrylate glue (Loctite 401). Finally, the square rubber ring layer was attached to the tungsten base around the PMN-28PT sample to support the fixation. Figure 9c shows a photograph of the completed compressive vector sensor after two flexible cables were attached using solder.

### 3.2. Characterization of the Prototype Vector Sensor

**Figure 10:**
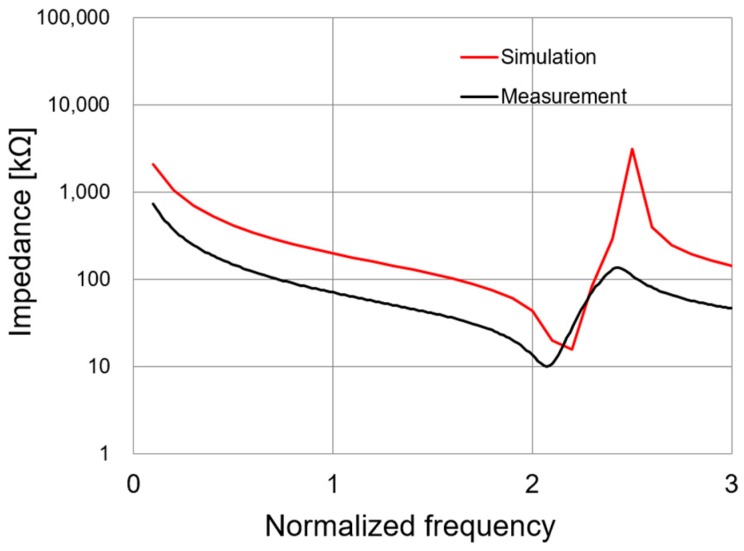
A comparison of the simulated and experimental vector sensor impedance spectra.

The impedance spectrum and directivity pattern of the fabricated accelerometer were measured to verify the characteristics and validity of the designed vector sensor. Figure 10 shows the impedance of the prototype vector sensor and the FEA simulation model as a function of frequency in the air (oscillation level: 500 mV). The electrical impedance spectrum of the prototype sensor was measured with an impedance/gain phase analyzer (HP4294A, Agilent, Santa Clara, CA, USA) with a 42941A probe kit (Keysight, Santa Clara, CA, USA).

Good agreement was observed, and both measurements exhibited a resonant frequency above 2*f*_0_. Generally, vector hydrophone sensors should be designed with a resonant frequency above the intended operating frequency range so that the sensitivity is reasonably flat across the intended frequency spectrum. Accordingly, the performance of the proposed vector sensor designed here meets the operating frequency requirements (i.e., <*f*_0_).

**Figure 11:**
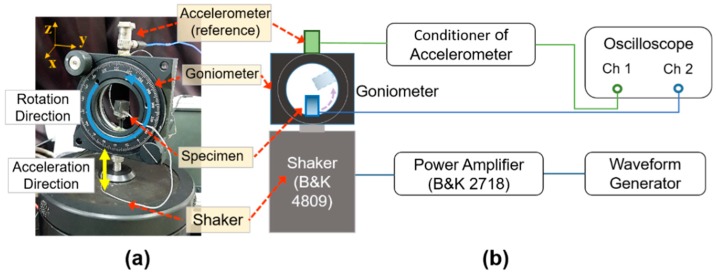
(**a**) A photograph and (**b**) schematic diagram of the experimental setup for directivity measurement.

**Figure 12:**
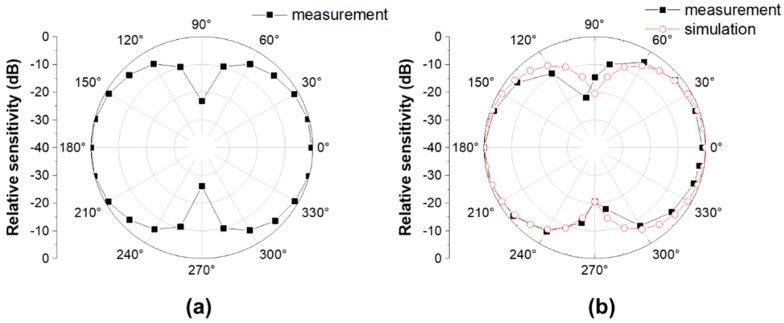
(**a**) The dipole beam pattern of the fabricated vector sensor measured at 0.01*f*_0_. (**b**) A comparison of the simulated and experimental dipole beam patterns of the vector sensor at 0.1*f*_0_.

The vector sensor directivity was characterized using a shaker (type 4809; Brüel & Kjaer, Nærum, Denmark) and goniometer, as shown in Figure 11. The shaker was driven by a waveform generator (33500B; Keysight, Santa Rosa, CA, USA) and amplifier (type 2718; Brüel & Kjaer) to excite the vector sensor with various frequencies and acceleration levels. The goniometer, fabricated vector sensor, and commercial accelerometer (J352C33; PCB Piezotronics, Depew, NY, USA) were mounted on top of the shaker so that the center axis of each component was coincident. While the shaker was excited under the sinusoidal excitation at 0.5 G [1 G = 9.8 m/s^2^] acceleration in the z-direction (yellow arrow) monitored by the commercial accelerometer, the vector sensor attached to the inner circle of the goniometer was rotated count clockwise (blue arrow), as shown in Figure 11a. In order to obtain a beam pattern, the output voltages of the vector sensor were measured with an oscilloscope at each rotation angle (*θ*) of the goniometer in 15°. During the measurements, the vector sensor was driven at 0.01*f*_0_ and 0.1*f*_0_. The receiving beam pattern derived in Equation (2) with an output signal from the sensor clearly exhibits an apparent dipole mode which represents the ability of the sensor to determine the direction of an incoming acoustic signal. A maximum sensitivity difference of 23 dB was found between 90° (or 270°) and 0° (or 180°), as shown in Figure 12a. The dipole directivities of the measured and simulated vector sensor were in good agreement, as shown in Figure 12b.

**Figure 13:**
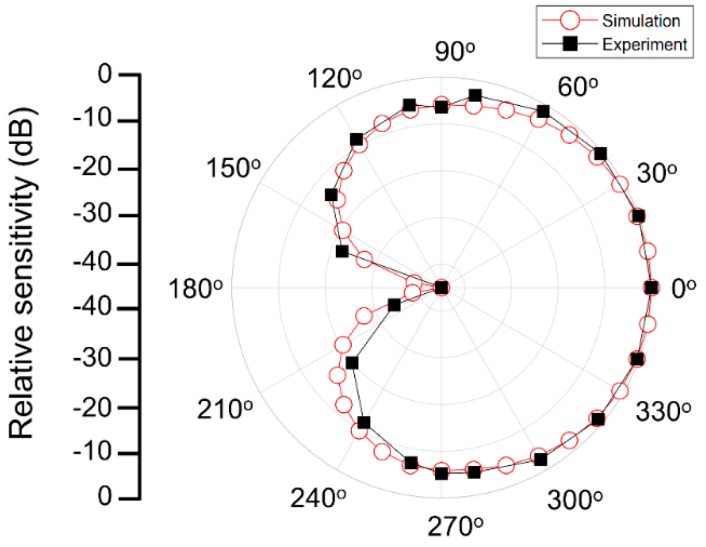
A synthesized 2-D cardiac beam pattern comparison between the FEA simulation and fabricated vector sensor.

The polar response of the compressive single crystal sensor indicates that the axial sensor can be used to produce a cardioid pattern when combined with an omnidirectional hydrophone, as shown in Figure 13. The simulated omnidirectional hydrophone beam pattern from Figure 6 was used to calculate the cardioid beam pattern. The cardioid patterns based on FEA simulation and measurement experiment were found to be in good agreement. This indicates that the vector sensor studied here has the potential to be integrated with an omnidirectional hydrophone to utilize as a vector hydrophone.

## 4. Conclusions

A small compressive accelerometer vector sensor comprising a PMN-28PT single crystal and tungsten seismic mass was shown successfully to form a highly directional dipole pattern that could be used for underwater source detection. FEA simulation results were used to determine the optimal device dimensions of a vector sensor. The RVS of the vector sensor was improved by 8 dB by changing the dimensions of the vector sensor. In addition, the characteristics of the dipole beam pattern, which were a function of the geometric variations of the piezoelectric single crystal, were analyzed. The vector sensor was fabricated with optimized dimensions and was found to form a well-defined dipole pattern that could be used to identify the directivity of an acoustic source at the frequency of interest. The performance of the fabricated sensor was consistent with the simulated device. For simulation, a 3-D dipole pattern was constructed by adding an orthogonal rotation based on the FEA model to show the possibility to identify the directivity of an acoustic source in 3-D space.

## Figures and Tables

**Table 1:** Material properties of the PMN-28PT [22].

**Elastic Compliance Constants (*S*) [10^−12^ m^2^/N]**	$S_{11}^{E}$	41.4	**Piezoelectric Strain Constants (*d*) [10^−12^ C/N]**	*d*_31_	−549
	$S_{12}^{E}$	−22		*d*_33_	1282
	$S_{13}^{E}$	−19.4		*d*_15_	169
	$S_{33}^{E}$	47.5	**Dielectric Constants (*ε*)**	$\varepsilon_{11}^{T}/\varepsilon_{0}$	1821
	$S_{44}^{E}$	15.1		$\varepsilon_{33}^{T}/\varepsilon_{0}$	4841
	$S_{66}^{E}$	24.9	**Density (*ρ*) [kg/m^3^]**	*ρ*	8000

**Table 2:** Material properties of the tungsten.

Young’s Modulus [GPa]	Density [kg/m^3^]
360	17,800

**Table 3:** Dimensions of the accelerometer.

Material	PMN-28PT		Tungsten	
**Parameter**	***w_s_***	***t_s_***	***w_m_***	***t_m_***
**Dimension [mm]**	2.5–5	0.1–1	5–10	1–10

**Table 4:** Material properties of the PZT-4D [24].

**Elastic Compliance Constants (*S*) [10^−12^ m^2^/N]**	$S_{11}^{E}$	13.3	**Piezoelectric Strain Constants (*d*) [10^−12^ C/N]**	*d*_31_	−135
	$S_{12}^{E}$	−4.76		*d*_33_	315
	$S_{13}^{E}$	−6.2		*d*_15_	550
	$S_{33}^{E}$	16.8	**Dielectric Constants (*ε*)**	$\varepsilon_{11}^{T}/\varepsilon_{0}$	1610
	$S_{44}^{E}$	42		$\varepsilon_{33}^{T}/\varepsilon_{0}$	1450
	$S_{66}^{E}$	36.1	**Density (*ρ*) [kg/m^3^]**	*ρ*	7600

## References

[1] Lu F., Milios E., Stergiopoulos S., Dhanantwari A. (2003). New towed-array shape-estimation scheme for real-time sonar systems. IEEE J. Ocean. Eng..

[2] Heidemann J., Li Y., Syed A., Wills J., Ye W. Research challenges and applications for underwater sensor networking. Proceedings of the IEEE Wireless Communications and Networking Conference 2006.

[3] Gabrielson T.B., Gardner D.L., Garrett S.L. (1995). A simple neutrally buoyant sensor for direct measurement of particle velocity and intensity in water. J. Acoust. Soc. Am..

[4] Bastyr K.J., Lauchle G.C. (1993). Development of a velocity gradient underwater acoustic intensity sensor. J. Acoust. Soc. Am..

[5] Sherman C.H., Butler J.L. (2007). Transducers and Arrays for Underwater Sound.

[6] Schau H.C., Robinson A.Z. (1987). Passive source localization employing intersecting spherical surfaces from time-of-arrival differences. IEEE Trans. Acoust. Speech Sig. Process..

[7] Silvia M.T., Richards R.T. A theoretical and experimental investigation of low-frequency acoustic vector sensor. Proceedings of the Oceans 2002.

[8] Lim Y., Joh C., Seo H., Kim J., Roh Y. (2014). Design and fabrication of a multimode ring vector hydrophone. Jpn. J. Appl. Phys..

[9] Kalgan A., Bahl R., Kumar A. (2015). Studies on underwater acoustic vector sensor for passive estimation of direction of arrival of radiating acoustic signal. Indian. J. Geo-Mar. Sci..

[10] Spain G.L.D., Hodgkiss W.S., Edmonds G.L. (1991). The simultaneous measurement of infrasonic acoustic particle velocity and acoustic pressure in the ocean by freely drifting Swallow floats. IEEE J. Ocean. Eng..

[11] Abdi A., Guo H., Sutthiwan A. new vector sensor receiver for underwater acoustic communication. Proceedings of the Oceans 2007.

[12] Felisberto P., Santos P., Jesus S.M. Tracking source azimuth using a single vector sensor. Proceedings of the 2010 Fourth International Conference on Sensor Technologies and Applications.

[13] Shipps J.C., Deng K. A miniature vector sensor for line array applications. Proceedings of the Oceans 2003.

[14] Beranek L.L., Mellow T. (2012). Acoustics: Sound Fields and Transducers.

[15] Pyo S., Kim J., Kim H., Roh Y. (2018). Development of vector hydrophone using thickness-shear mode piezoelectric single crystal accelerometer. Sens. Actuators A.

[16] Butler J.L., Sherman C.H. (2016). Transducers and Arrays for Underwater Sound.

[17] McConnell J.A., Jensen S.C., Rudzinsky J.P. Forming first- and second-order cardioids with multimode hydrophones. Proceedings of the Oceans 2006.

[18] Agarwal A., Kumar A., Aggarwal M., Bahl R. Design and experimentation with acoustic vector sensors. Proceedings of the 2009 International Symposium on Ocean Electronics (SYMPOL 2009).

[19] Kim K., Gabrielson T.B., Lauchle G.C. (2004). Development of an accelerometer-based underwater acoustic intensity sensor. J. Acoust. Soc. Am..

[20] Angleton P.A., Hayer J.R. (1969). Ceramic transducer elements and accelerometers utilizing same. US Patent.

[21] Tims A.C., Davidson R.L., Timme R.W. (1975). High sensitivity piezoelectric accelerometer. Rev. Sci. Instrum..

[22] Kim J., Joh C., Roh Y. (2013). Evaluation of all the Material Constants of PMN-28%PT Piezoelectric Single Crystals for Acoustic Transducers. Sens. Mater..

[23] Lurton X. (2002). An Introduction to Underwater Acoustics.

[24] Efunda Piezo Data: PZT-4D. https://www.efunda.com.

